# “We want more”: perspectives of sarcopenic older women on the feasibility of high-intensity progressive resistance exercises and a whey-protein nutrition intervention

**DOI:** 10.3389/fnut.2023.1176523

**Published:** 2023-09-07

**Authors:** Reena K. Vijayakumaran, Robin M. Daly, Vina P. S. Tan

**Affiliations:** ^1^Department of Rehabilitation and Sports Science, Faculty of Health and Social Sciences, Bournemouth University, Bournemouth, United Kingdom; ^2^Institute for Physical Activity and Nutrition, School of Exercise and Nutrition Sciences, Deakin University, Geelong, VIC, Australia; ^3^Exercise & Sports Science, School of Health Sciences, Universiti Sains Malaysia, Kubang Kerian, Malaysia

**Keywords:** sarcopenia, older adults, resistance exercises, whey-protein, qualitative, pilot, randomized-trial

## Abstract

This qualitative study is nested within a 12-week pilot randomized-controlled, two-arm trial involving high-intensity progressive resistance training (PRT) or PRT with a multi-nutrient, whey-protein supplementation (PRT+WP) in sarcopenic older adults (trial registration no: TCTR20230703001). The aim was to investigate sarcopenic participants' perceptions and barriers to this multi-modal intervention strategy that may accelerate “real-world” implementation. Eighteen older adults (one man) with possible sarcopenia were invited to join the study, of whom 16 women were randomized to a thrice-weekly PRT (*n* = 8) program (80% of 1-repetitive maximum, six resistance band exercises) only or PRT plus daily weekday milk-based WP supplementation (PRT+WP, *n* = 8). Muscle strength (handgrip and 5-times sit-to-stand), mass (dual-energy X-ray absorptiometry), performance (Short Physical Performance Battery and stair ascent-descent), and nutrition status (Mini Nutritional Assessment) were assessed for changes. We randomly selected eight women for the semi-structured interview. Post-intervention, eight (50%) women were sarcopenia-free, six (38%) remained in possible sarcopenia, one (6%) improved to sarcopenia, and one (6%) deteriorated from possible to severe sarcopenia. There were no significant between-group differences, but significant within-group improvements (*p* < 0.05) were detected for handgrip strength (PRT+WP 5.0 kg, *d* = 0.93; PRT 6.1 kg, *d* = 0.55), 5-times sit-to-stand time (PRT 2.0 s, *d* = 1.04), nutrition score (PRT+WP 3.44, *d* = 0.52; PRT 1.80, *d* = 0.44), and stair ascent time (PRT+WP 0.97 s, *d* = 0.77; PRT 0.75 s, *d* = 0.97). Our thematic analyses identified four main themes, namely, (1) perceived benefits, (2) sustaining behavior changes, (3) challenges in participating, and (4) improved wellbeing. Participants expressed how they initially were skeptical and doubted that they could complete the exercises or tolerate the milk-based WP supplements. However, they reported positive experiences and benefits felt from strength gains, increased confidence, and better physical abilities. Participants were surprised by the zero adverse effects of WP supplements. The women wanted more nutritional information and structured, guided exercise programs and suggested a community-based implementation. In conclusion, our findings showed PRT was well received and may support reduced risks of sarcopenia. No added benefits were seen with the addition of WP supplementation, but a larger sample is required to address this question. Overall, older (previously sarcopenic) Malay women indicated that they want more multi-modal programs embedded in their community.

## 1. Introduction

Sarcopenia is an age-related, progressive, and generalized skeletal muscle disorder involving a loss of muscle mass, strength, and function that is associated with increased adverse outcomes including falls, frailty, fractures, and premature mortality in older adults (1). The overall prevalence of sarcopenia is estimated to be 10% worldwide in those aged at least 60 years and above, with some reports of a higher prevalence among Asians than non-Asian countries (2). Sarcopenia prevalence is further confounded by the different cut-points adopted by several key working groups such as the European Working Group on Sarcopenia in Older People, the Asian Working Group for Sarcopenia, and the International Working Group on Sarcopenia that may be region-specific in their application (2). Research in Malaysia on sarcopenia is still limited, but one study among 393 adults aged 60 years and over reported an overall prevalence of 33.6% (3). The prevalence of sarcopenia (and its components) is related to exorbitant healthcare costs (4), and thus the maintenance of muscle mass, strength, and function is critical to avoid the financial burden related to home-assisted living and institutionalized care in older people (5). With no known pharmaceuticals available to treat sarcopenia (6, 7), strategies to increase muscle mass, strength, and function focus on modifiable lifestyle behaviors and practices that have been shown to be effective.

Adequate nutrition, such as high protein intake, sufficient calories, adequate omega n-3 fatty acid intakes, and regular exercise, is widely recommended for people diagnosed with sarcopenia (8). More specifically, current guidelines for the treatment and prevention of sarcopenia recommend muscle strengthening or progressive resistance training (PRT) along with an adequate intake of protein and sufficient vitamin D (9–12). In sarcopenic older adults, PRT exercises are safe and effective to improve muscle health, and the addition of protein with PRT may provide some additional, albeit modest, benefits to muscle mass and strength (13, 14). Despite these benefits, adherence to PRT among community-dwelling older adults remains low (<10–15%) (15). Older people's uptake and engagement with such programs can be influenced by a range of behavioral factors, such as motivation and personal beliefs, as well as environmental factors, including the availability of public transport, cost, and the location and type of exercise venues (16).

Despite the positive responses from previous studies evaluating the effectiveness of exercise and/or nutritional intervention on sarcopenia outcomes (13, 14), there has been little research performed in Malaysia. A large proportion of older adults in Malaysia are physically inactive, with 30% reporting not engaging in any regular activity (17). The Malaysian community comprises different ethnicities, body sizes and compositions, food cultures, and physical activity habits compared to other populations (18, 19). To date, no studies have explored the beliefs and perceptions of older Malaysian adults with or at risk of sarcopenia about participating in a multi-modal lifestyle intervention incorporating high-intensity PRT with a whey-protein-based nutrition supplemental drink (WP). This is important to inform our understanding of the behaviors (motivation and barriers) and decision-making on sarcopenia in this population to inform future initiatives to prevent and treat this disease and support sustainable behavior change. Therefore, this pilot study aimed to identify the key facilitators and barriers for older Malaysian adults with sarcopenia to participate in a 12-week PRT program with or without the consumption of a WP nutritional supplement.

## 2. Materials and methods

### 2.1. Study design

This is a qualitative study nested within a 12-week randomized-controlled, two-arm trial involving PRT alone or combined PRT+WP supplementation. This research was approved by the Human Research Ethics Committee of Universiti Sains Malaysia (USM/JEPeM/18090405).

### 2.2. Participants

We reached older Malaysian adults through online advertisements and community-focused areas such as mosques, community halls, and private health facilities. Using the Asian Working Group for Sarcopenia 2019 (AWGS2) (20) guidelines, we initially screened for possible sarcopenia and included participants with a handgrip strength (HG) of <28 kg (men) or <18 kg (women) using their dominant arm and/or a five-times sit-to-stand time (5-STS) of 12 s and more. HG test was conducted using a hand-held dynamometer (Jamar, USA), where the participants were seated and their arms bent at 90° at the elbow. Participants were given verbal encouragement during each test and were instructed to squeeze as hard as they could and alternate arms to complete the test three times per arm. The highest value (in kg) obtained was used as the maximal HG strength. Participants who were identified as having possible sarcopenia, physically inactive (did not achieve 150 min/week of physical activity), and did not participate in any structured exercise programs in the past 6 months were invited to join the study. Participants who provided informed written consent later completed the rest of the assessments at the Universiti Sains Malaysia, Health Campus, Kelantan.

### 2.3. Physical measures

The following measures were assessed at baseline and post-intervention: Height measurements were conducted three times using a stadiometer (SECA, Germany), and participants' weight was measured using a digital weighing scale (Omron, USA). Appendicular skeletal muscle mass (ASM, kg/m^2^) was assessed from a total body dual-energy X-ray absorptiometry scan (DXA, Discovery A, Hologic, USA), with low ASM classified as <7.0 kg/m^2^ for men and <5.4 kg/m^2^ for women (20). Physical performance was assessed using the Short Physical Performance Battery (SPPB) that includes balance (side-by-side, semi-tandem, and tandem), 5-STS, and a 4-m gait assessment (21), with impaired physical performance classified as a SPPB score of ≤9. Sarcopenia classifications followed the possible sarcopenia (low grip strength and/or slow 5-STS time), sarcopenia (possible sarcopenia or low SPPB plus low ASM), and severe sarcopenia (presence of all three low measures) definitions from AWGS2 (20). Participants also completed the Mini Nutritional Assessment (MNA) that includes measurements of left-side mid-arm and calf circumferences (22). Additionally, we assessed stair ascent and descent time using a standardized set of 10 steps, whereby participants were asked to walk as quickly up or down the stairs as possible, with the time recorded to the nearest 0.1 s (23).

### 2.4. Intervention

A total of 16 participants (women) were randomized to the PRT+WP (*n* = 8) or PRT alone (*n* = 8) groups. A research assistant used simple computer randomization to allocate the participants into their groups. Participants in the PRT+WP group had a daily weekday serving of 100% whey protein concentrate (Enprovis Plus, Aegisu Sdn. Bhd.) that consists of 237 kcal, 15.1 g protein, 250 mg calcium, 3.3 mg iron, 4.0 μg vitamin D, and other micronutrients that cover 14–93% of the daily recommended nutrient intake for those aged 60 years and above. The full nutrition composition is provided in Supplementary material 1. The supplement was consumed as part of their morning breakfast on non-exercise days or within a 30-min window after their exercise sessions.

All participants were prescribed a high-intensity PRT program that could be conducted at home thrice a week. Prior to commencing PRT, a qualified Master of Science in Exercise Science graduate assessed baseline muscle strength and taught proper exercise techniques and warm-up and cool-down activities to all participants. Participants had their blood pressure and heart rate checked using an automated blood pressure device (Omron, Japan) after sitting quietly for about 3 min to rule out uncontrolled hypertension. Under supervision, participants practiced the exercise movements without any resistance bands twice a week for 2 weeks before the intervention period started. There were six resistance band exercises, namely, (i) squats, (ii) gluteal kickbacks, (iii) seated leg extensions, (iv) standing chest press, (v) standing diagonal pull apart, and (vi) seated row. To assess 1-RM, participants were given a resistance band to complete 10–12 repetitions of each exercise without losing their form or technique. If participants could complete 12 repetitions easily, a thicker (higher) resistance band was provided, and the same exercise was conducted after a 2–3 min rest until the suitable resistance band was identified as the participant's 1-RM load. If participants struggle to complete eight reps adequately, they will be given a thinner (lower) resistance band. As the same resistance band can provide varied tension depending on the tautness, participants were taught to hold their resistance bands in a straight line, with no slacking or excess pull, at the starting position of each exercise. For the first 2 weeks of familiarization, participants used light resistance bands, performing two sets of 10–15 repetitions for each of the six exercises. Exercises increased progressively, with adjustments occurring at weeks 2, 4, and 8. At each progression check, only one component was increased, such as repetitions or adding an additional set. If participants reached three sets of 10 reps with the current resistance band, the resistance band was changed to the next higher resistance band with a reassessment of the number of reps and/or sets to be conducted. Participants achieved and maintained their individualized equivalent of 75–80% of 1-RM throughout the rest of the intervention period and progressed to three sets of 8–10 repetitions for about 4 weeks before the intervention concluded. The resistance bands, length 2.0 m × width 150 mm (Exercise Band, Top Glove, Malaysia), have a variable thickness of 0.15–0.45 mm (increments of 0.05 mm), and the maximal band thickness used by participants was 0.20 mm, or the equivalent of 4.3–4.7 kgf at 300% extension (manufacturer's information). All exercise prescriptions and progressions were conducted in person and then monitored through weekly inquiries by the research team that checked on the completion of exercise and supplement intake based on the participants' self-report for that week. During the weekly calls, participants were also asked to report any adverse events related to the prescribed exercise or nutritional supplement.

### 2.5. Semi-structured interviews

Upon completing the 12-week intervention, using a purposive sampling method, a total of 8 of the 16 participants participated in a semi-structured interview, achieving the data saturation concept. Data saturation was reached when the information from participants no longer offered new insights and views that others had not mentioned (24). Each interview lasted approximately 30–40 min using an interview guide (Supplementary material 2). Interviews were conducted over the phone at the participant's convenience as this was during the COVID-19 pandemic lockdown in Malaysia and face-to-face interviews were not possible. Interviews were recorded in a local Malay dialect, translated to English, and transcribed by the same trained research assistant. There were no pilots or repeat interviews. Our qualitative researcher counter-checked the translation and transcription and the field notes made during the interviews. The NVivo software, version 12 (QSR International, Australia), was used by the researcher to organize, explore, integrate, and finally interpret the data. Thematic analysis using the six phases by Braun and Clarke (25) provided rigorous and trustworthy data analysis and interpretation. Data were presented according to emerging themes that were discussed and agreed upon by two researchers. Qualitative reports were guided by the consolidated criteria for reporting qualitative research (COREQ) checklist (26).

### 2.6. Statistical analyses

Statistical analyses of the quantitative measures were conducted using SPSS Statistics for Windows, version 26 (Armonk, NY: IBM Corp.). The data met the assumption of normality, and baseline group differences were assessed using an independent *t*-test. Mean group differences for the change (post-intervention minus baseline values) were generated and compared using an independent *t*-test. Within groups, changes were assessed using a paired *t*-test, and assumptions were checked and matched for the inferential statistical analyses. Observed effect sizes of paired samples (within-group) were calculated using unbiased Cohen's *d* (post-intervention means minus baseline means, then divided by pooled standard deviations with correction), with an interpretation of the magnitude of *d* based on the following: 0.20 “small effect,” 0.50 “medium effect,” and 0.8 “large effect” (27). For the effect size of the net differences (between groups), *d* was calculated using the mean changes in PRT+WP minus the mean changes in PRT and divided by the pooled standard deviations of the respective means. Due to our modest sample size, the results and findings from the statistical analyses should be interpreted with caution. Statistical significance was set at a *p-*value of <0.05.

## 3. Results

### 3.1. Study participants, attrition, and adherence

**Figure 1:**
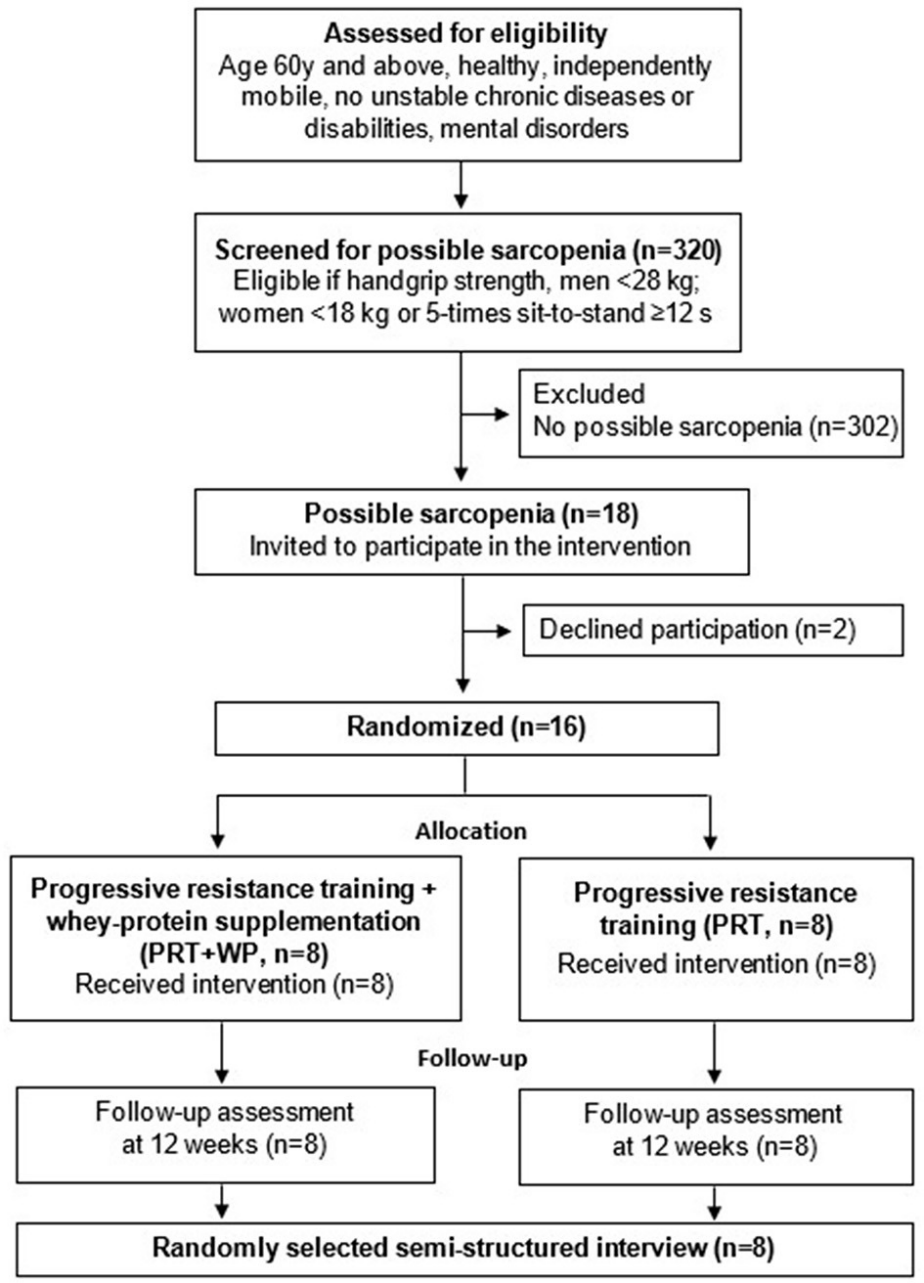
Flow diagram of participants through the study.

A total of 320 older adults were screened for this study, of whom 18 were identified as having possible sarcopenia (one man). Two participants (husband and wife) withdrew from the PRT group after citing personal concerns about attending measures and exercise intervention sessions. Notably, 16 women were randomly allocated to the PRT+WP (*n* = 8) or PRT (*n* = 8) groups. The recruitment and randomization process is displayed in Figure 1 based on the CONSORT extension to randomized pilot study guidelines (28).

Participants were community-dwelling, Malay women, aged (mean ± SD) 66.0 ± 3.1 years (range 61–72). Characteristics of the participants by PRT+WP and PRT group are displayed in Table 1. There were no significant differences between groups at baseline for all the measures, indicating randomization was achieved.

**Table 1:** Baseline characteristics (mean ± SD) in the progressive resistance training (PRT) plus whey protein supplementation (PRT+WP) and PRT only groups.

**Variables**	**PRT+WP**	**PRT**
n	8	8
Age (years)	66.6 ± 4.0	65.5 ± 1.9
Height (cm)	154.8 ± 5.8	151.6 ± 6.2
Weight (kg)	62.4 ± 10.0	55.9 ± 8.5
Body mass index (kg/m^2^)	26.4 ± 4.6	24.5 ± 4.3
Systolic blood pressure (mmHg)	126.0 ± 18.3	134.3 ± 17.8
Diastolic blood pressure (mmHg)	73.5 ± 11.5	71.9 ± 6.7
Resting heart rate (bpm)	78.6 ± 7.4	71.1 ± 11.1

Overall, 16 (100%) participants completed the intervention and the post-intervention measurements. We recorded that the participants had 90% adherence to the prescribed exercises and supplement intake throughout the intervention period. There were no reported adverse events from either the exercise or nutrition supplement.

### 3.2. Prevalence of sarcopenia

**Figure 2:**
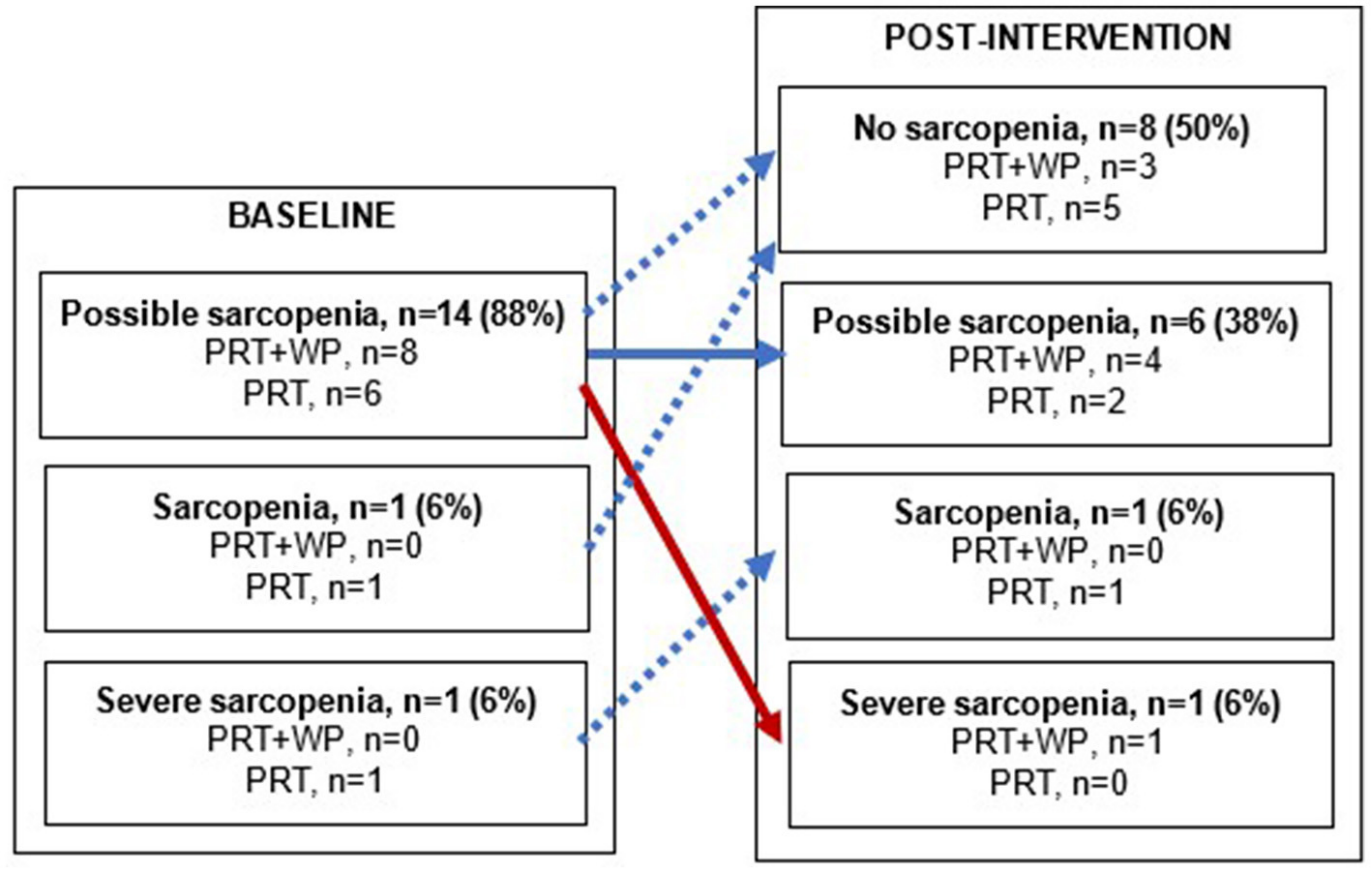
Changes in sarcopenia status from baseline to post-intervention within the progressive resistance training (PRT) plus whey protein supplementation (PRT+WP) and PRT-only groups. A solid blue arrow depicts no change in sarcopenia status, blue dashed arrows depict an improvement in sarcopenia status, and a red arrow depicts a decline in sarcopenia status.

At baseline, there were 14 women (88%) with possible sarcopenia (PRT+WP, *n* = 8; PRT, *n* = 6), one (PRT) classified as sarcopenic, and one with severe sarcopenia (PRT). As shown in Figure 2, at the end of the intervention, six (38%) women classified as having possible sarcopenia at baseline (PRT+WP, *n* = 4; PRT, *n* = 2) retained the same sarcopenia status, while nine (56%) women (PRT+WP, *n* = 3; PRT, *n* = 6) showed improvements in their sarcopenia status; one woman with possible sarcopenia progressed to severe sarcopenia.

### 3.3. Muscle strength, mass, and physical performance

There were significant mean changes for PRT+WP in handgrip strength (*p* = 0.004), stair ascent time (*p* = 0.03), and MNA scores (*p* = 0.009), while PRT also had significant mean changes for handgrip strength (*p* = 0.008), stair ascent time (*p* = 0.001), MNA scores (*p* = 0.003), and 5-STS time (*p* = 0.03) (Table 2). However, there were no significant between group net differences for the mean changes between PRT+WP and PRT for any of the sarcopenia components or physical outcomes (Table 2).

**Table 2:** Baseline (pre) and post-intervention (post) measures in the progressive resistance training (PRT) plus whey protein supplementation (PRT+WP, *n* = 8) and PRT only (*n* = 8) groups and the mean within group changes and net between group differences for the change along with the Cohen's d effect sizes.

	**PRT**+**WP**		**PRT**			
	**Mean** ±**SD or Mean change (95% CI)**	**d**	**Mean** ±**SD or Mean change (95% CI)**	**d**	**Net difference (95% CI)**	**d**
**Mid-arm circumference (cm)**						
Pre	29.0 ± 4.1		27.1 ± 4.1		-	
Post	30.2 ± 4.1		26.4 ± 4.2		-	
Δ (Post–Pre)	1.19 (−0.86 to 3.21)	0.07	−0.66 (−1.96 to 0.64)	0.04	1.85 (−0.33 to 4.03)	0.47
**Calf circumference (cm)**						
Pre	34.4 ± 4.7		32.1 ± 3.3		–	
Post	33.5 ± 3.4		32.2 ± 4.2		-	
Δ (Post–Pre)	−0.96 (−3.85 to 1.92)	0.06	0.18 (−1.18 to 1.53)	0.01	−1.14 (−4.03 to 1.76)	0.22
**ASM (kg/m** ^2^ **)**						
Pre	6.7 ± 0.7		6.2 ± 0.8		-	
Post	6.6 ± 0.8		6.3 ± 0.9		-	
Δ (Post–Pre)	−0.15 (−0.30 to 0.01)	0.24	0.14 (−0.12 to 0.40)	0.18	−0.29 (−0.56 to −0.01)	0.58
**Handgrip strength (kg)**						
Pre	15.8 ± 1.8		15.1 ± 0.4		–	
Post	20.8 ± 2.7		21.3 ± 4.6		-	
Δ (Post–Pre)	5.0 (2.2 to 7.8)^*^	0.93	6.2 (2.2 to 10.1)^*^	0.55	−1.1 (−5.5 to 3.3)	0.14
**5-times sit-to-stand (s)**						
Pre	12.9 ± 2.9		11.4 ± 1.3		-	
Post	11.6 ± 2.9		9.4 ± 1.4		-	
Δ (Post–Pre)	−1.3 (−3.4 to 0.9)	0.14	−2.0 (−3.8 to −0.2)^*^	1.04	0.70 (−1.78 to 3.37)	0.17
**SPPB score**						
Pre	9.1 ± 1.6		9.8 ± 1.3		-	
Post	9.3 ± 1.4		10.0 ± 0.5		-	
Δ (Post–Pre)	0.20 (−1.17 to 1.42)	0.05	0.25 (−0.62 to 1.12)	0.25	−0.13 (−1.56 to 1.31)	0.05
**Stair ascent time**						
Pre	8.4 ± 0.9		8.1 ± 0.8		-	
Post	7.4 ± 1.3		7.4 ± 0.9		-	
Δ (Post–Pre)	−0.97 (−1.80 to −0.10)^*^	0.77	−0.75 (−1.0 to −0.45)^*^	0.97	−0.23 (−1.06 to 0.61)	0.16
**Stair descent time**						
Pre	8.8 ± 1.6		9.1 ± 1.5		-	
Post	7.6 ± 1.1		8.7 ± 2.0		-	
Δ (Post–Pre)	−1.19 (−2.51 to 0.12)	0.57	−0.40 (−1.13 to 0.34)	0.12	−0.79 (−2.16 to 0.57)	0.32
**MNA score**						
Pre	24.8 ± 3.2		25.6 ± 2.2		-	
Post	28.2 ± 1.6		27.3 ± 1.7		-	
Δ (Post–Pre)	3.4 (1.2 to 5.7)^*^	0.52	1.8 (0.8 to 2.7)^*^	0.44	1.60 (−0.56 to 3.93)	0.44
**Well-nourished/Risk of malnutrition**						
Pre	4/4		6/2		-	
Post	7/1		8/0		-	

Pre and post-intervention values are mean ± SD; mean within group changes and net different mean (95% CI). *d*, Cohen's effect size with correction; ASM, appendicular skeletal muscle mass; SPPB, Short Physical Performance Battery; MNA, Mini Nutrition Assessment. ^*^p < 0.05, within-group change.

### 3.4. Nutritional status

The PRT+WP group significantly improved their MNA scores (*p* = 0.009) as did the PRT (*p* = 0.003). Although the PRT+WP group's improvement was nearly double the gains compared to PRT, there were no significant differences between the two groups (Table 2).

### 3.5. Perceptions on PRT and WP

**Figure 3:**
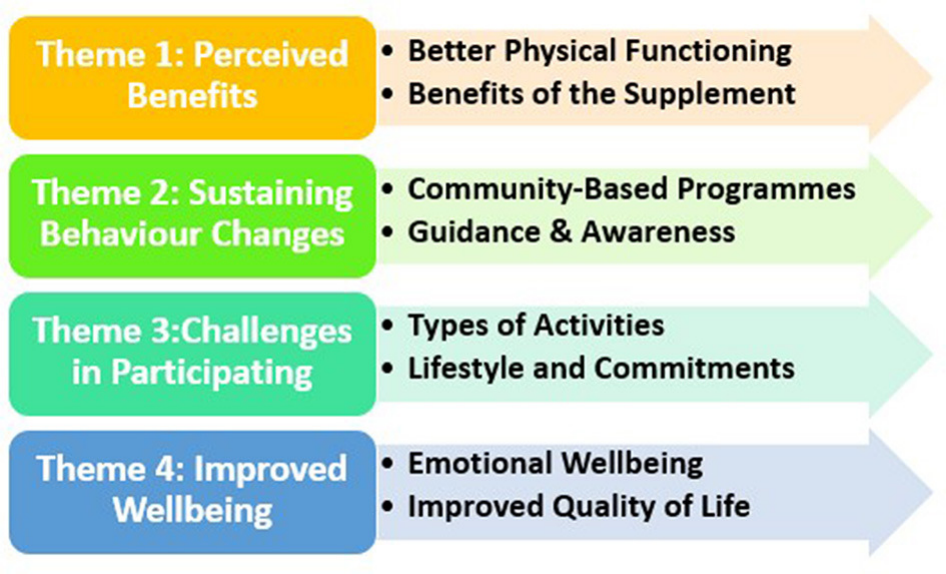
Themes on the benefits and challenges of participating in a 12-week exercise and nutrition supplementation intervention based on the perceptions of sarcopenic older women.

Eight women (5 PRT+WP, 3 PRT) aged 66.6 ± 4.0 years (ranging from 61 to 72) from the completed intervention group took part in the semi-structured interviews. Thematic analysis was used to identify benefits and challenges as perceived by the participants. In total, there were four main themes with sub-themes identified from the data (Figure 3). The data presented are representative of the coding used and the overall findings of the research.

#### 3.5.1. Theme 1: Perceived benefits

Perceived benefits were the most common aspect discussed by the participants, focusing on both the exercise and the high-protein whey-based nutritional supplement. Before starting the intervention, participants were informed about their sarcopenic condition. Perceived benefits were the key driver for them to participate in the study as they were looking for ways to manage and address their condition. Thus, there was no hesitation to participate.

##### 3.5.1.1. Better physical functioning

The benefits derived from the progressive resistance exercise were mainly regarded as a positive outcome as they were beginning to feel better throughout the intervention, especially upon completion. All women were physically inactive before participating in the exercise program and welcomed the idea of incorporating regular physical activity into their daily routine as a result of participating in the study. They also noted that exercising was particularly “scary,” and most of them were worried about the impact—falling or hurting themselves physically. However, once they started training, their self-confidence increased.

“*Before, I did not exercise. I used to be tired, but after participating in this research, I feel more energetic. I am getting fitter! My daily movement has become smoother too.” (Participant 1, 72 years)*

“*At first, I was reluctant and was unable to do all the exercises. I was scared that I will fall. I feel better physically now!” (Participant 2, 64 years)*

“*It was scary for me before starting but I went on to do the exercises because it restored my energy, less muscles pain and I learnt how to exercise properly. After exercising, I felt much more comfortable with my body (laughed)”. (Participant 3, 68 years)*

“*I feel my knees are much better now, l can use squatting toilet now. Happy!” (Participant 5, 61 years)*

“*It was difficult at the beginning but the longer I did, the easier it was. I am excited to do this exercise every day now as I feel better physically.” (Participant 8, 72 years)*

##### 3.5.1.2. Benefits of the nutrition supplement

All women enrolled in the study did not consume any protein-based supplements before joining the intervention. Five women that were interviewed were randomized to receive the nutritional supplement. They perceived themselves as lacking awareness and knowledge on nutrition for sarcopenia and healthy aging. This highlights that more needs to be done to advocate the benefits and importance of high protein-based diets or supplements for older adults with sarcopenia. Some participants indicated that they were worried that it might be similar to past experiences of ingesting milk and milk-based products, which had resulted in bloating and other adverse reactions. However, their overall experience with regard to consuming the 100% whey-based supplemental drink was very positive, and they liked the taste and mouthfeel. There were no adverse effects of the supplement based on self-reported data. More importantly, they stated that they did not experience the similar discomforts they had previously related to milk ingestion. They were ready to include the nutrition supplement in their daily diet as they perceived it to be beneficial for their health.

“*The milk was very delicious, and I was full after consuming it. As a result, I did not snack much and was able to control my rice consumption. Both milk and exercise helped to improve the body. I feel healthier now!” (Participant 3, 68 years)*

“*I mixed the milk with chocolate powder at first as normally I get nauseated and bloated tummy if I drink milk. I was surprised it was not like other supplements. The taste was good, and I recommend this product. People like me should be advised to drink this every day.” (Participant 7, 64 years)*

“*I don't drink milk much, but I started to take this supplement. It was delicious, and I can continue to drink it as there were no side effects.” (Participant 5, 61 years)*

#### 3.5.2. Theme 2: Sustaining behavior changes

The readiness to change behavior was evident in various aspects discussed with the women. They wanted to change their behavior after learning about sarcopenia and experiencing the intervention. In the long term, they want to improve their lifestyle by exercising and enhancing their knowledge and awareness on nutrition, physical activity, and sarcopenia. The impact of knowing that they were sarcopenic may have influenced their perception, but they were keen to change their behavior related to health. A group or community-based approach rather than an individual approach was perceived as more successful in sustaining their behavior change. Women recruited into this study had responsibilities and routines at home that they were used to, but they willingly accommodated the changes as part of the intervention.

##### 3.5.2.1. Community-based program

There was a preference for the training location to be somewhere where people with similar conditions could meet and exercise together, such as a community-based activity center. They were willing to allocate a certain time of the day to focus on exercising but preferred to attend a specific location to participate in a group activity.

“*The exercise sessions must be offered to others with the condition… sarcopenia. I felt more energetic and will be useful for others.” (Participant 4, 66 years)*

“*There should be similar exercise sessions for older women in a hall or community center. I am happy to join and will ask my friends to join. We will enjoy exercising together.” (Participant 1, 72 years)*

“*The exercise sessions should be offered beyond the research in groups. I would like to meet others and exercise together!” (Participant 6, 66 years)*

##### 3.5.2.2. Guidance and awareness

Women felt that there was a lack of information on exercise and nutrition for sarcopenia and older adults' health in Malaysia. They learned about nutrition as part of their involvement in the research but would like to have more information. Once they were diagnosed with sarcopenia, they valued greatly any information, especially on nutrition, to ensure their condition did not deteriorate. Dissemination of evidence-based information and guidelines through effective platforms and channels is needed, especially for this age group in Malaysia.

“*I did not know much about both exercising and nutrition before joining the research.” (Participant 1, 72 years)*

“*I would like to know about exercising, proper food and nutrition for my condition and age as there is not much info out there.” (Participant 7, 64 years)*

“*I would like some guidance on both nutrition and exercising, especially for my condition. I want to feel better, and sure I will take care better if I have the information.” (Participant 8, 72 years)*

“*To be healthier long-term and beyond the research, more information will be valuable and much appreciated.” (Participant 2, 64 years)*

#### 3.5.3. Theme 3: Challenges in participating

Study participants highlighted several challenges in the prescribed exercises but perceived that the benefits outweighed these challenges at the end of the intervention. Exercise itself was perceived as a barrier before the intervention. However, the challenges/barriers became their motivation to be more physically active.

##### 3.5.3.1. Types of activities

Certain exercises were challenging to some of the women: chest press, seated leg extensions, and squatting exercises were mentioned. However, self-determination was observed as there were many situations where the exercises were difficult, but all the participants continued and completed most of their prescribed program successfully. At times, they reduced the exercise repetitions but did not stop the exercises. In terms of perceived benefits, how they felt both physically and emotionally upon completing the activities was the main reason for their strong commitments. They felt fitter, were able to move more, felt stronger, and were more confident in doing their daily activities.

“*Some exercises, such as the chest strengthening, was difficult at the start, but I slowed down at first and later continued as I was able to do more, felt less pain.” (Participant 5, 61 years)*

“*It was difficult to do the squatting exercise. I stopped when it was painful, but by end of the study, I was able to do it! Felt really happy!” (Participant 6, 66 years)*

“*The chest strengthening was not easy for me, but others were relatively easy. I took more breaks in between but made sure I completed the exercises.” (Participant 4, 66 years)*

“*I felt my muscles were stronger after exercising, and I was able to do my activities like walking and household work better.” (Participant 1, 72 years)*

##### 3.5.3.2. Lifestyle and commitments

Lack of time or their current lifestyle was a concern before they started, but they were willing to change their lifestyle to accommodate the activities prescribed during the intervention. This was attributed to their fewer responsibilities due to their age and role at home (retired, not much work to do at home, and no childcare tasks). Therefore, they had a high level of commitment and included the exercises in their usual routine. Participants understood that behavior change was one of the key elements to ensure the sustainability of their actions, especially in the long term. Focusing on activities that emphasize healthy aging was welcomed and appreciated. However, it was not clear and remains to be investigated if the participants continued to exercise beyond the intervention.

“*I allocated a specific time in the morning and evening, approximately 30–40 min. I was able to commit as I don't have many responsibilities and work around the house.” (Participant 7, 64 years)*

“*I usually set a time after the Asar prayers for about 30 min. The timing worked as I exercised after my daily prayer time. I had a routine that worked for me”. (Participant 6, 66 years)*

#### 3.5.4. Theme 4: Wellbeing

All participants wanted to continue with the intervention (exercise and consume the supplement). Improved quality of life and positive emotions were often described as favorable outcomes of the intervention.

##### 3.5.4.1. Emotional wellbeing

Negative emotions such as nervousness, anxiety, lack of confidence, and worry were common thoughts experienced by the women before the intervention. Perceived risk and discomfort were also sources of negative emotions prior to starting the intervention. However, with supervision and guidance from the research team, they were more confident and were able to adhere to the intervention. The perceived benefits were the main motivation as mentioned in other themes. Participants indicated that they were happier, cheerful, and satisfied, and had a more positive outlook on health, especially by the end of the intervention. Their feelings were attributed to the overall intervention and were not specific to either physical activities or supplementation. Positive emotions experienced also included improvements in quality of life and mental health. Emotions are not easily recognized or widely discussed, but they were evident in this study.

“*I was doubtful of myself…whether I can do the exercises, but now I am very happy and confident (laughed)!” (Participant 2, 64 years)*

“*Feeling happier and cheerful every day. I feel grateful for being a part of this research.” (Participant 3, 68 years)*

“*Before I started, I was quite nervous, mainly because I feared getting hurt. However, I was given adequate guidance on how to carry out the exercise. I am glad I took part, feeling more energetic every day now and feel blessed.” (Participant 7, 64 years)*

“*I am more positive now, very happy…maybe because I am more active (laughed).” (Participant 8, 72 years)*

##### 3.5.4.2. Improved quality of life

The overall perception of the intervention was positive as they expected and experienced an improved quality of life. They were more active physically, experienced less pain, and generally felt more positive about their health. All women commented on the improved quality of life due to the intervention. Their perception of quality of life was subjective but likely attributed to being physically active and learning new skills (exercising).

“*These types of activities should be provided to others too! It has improved how I work!” (Participant 1, 72 years)*

“*I have a better outlook of my life. I can move around more without pain. I am living better.” (Participant 3, 68 years)*

“*I feel the study is very good. It had a positive impact on my life, such program must continue!” (Participant 4, 66 years)*

## 4. Discussion

In this study of older sarcopenic Malay women, resistance training (with and without whey-protein-based nutritional supplementation) was associated with many positive perceptive and physical benefits. First, the home-based, high-intensity resistance training program was shown to be safe and effective to optimize muscle health (and even reversing sarcopenia) in older sarcopenic adults. Second, the following four main themes related to commencing and participating in a multi-modal exercise and nutrition intervention emerged: (1) perceived benefits, (2) sustaining behavior changes, (3) challenges in participating, and (4) improved wellbeing that supports the continuation of the exercise and nutrition supplementation. Sub-themes such as better physical functioning and perceived benefits of the nutritional supplementation indicated the positive impact of the intervention, while sub-themes such as preference for community-based programs and being guided and given awareness highlighted their intention to continue with such activities. Challenging activities and how they overcome them were also shared, which are important to inform future implementation. Third, through the interviews, participants' emotional experiences started with fearfulness and doubt about their ability to conduct PRT and consume the WP supplement, but having no adverse effects from both components, the emotions were replaced by a strong sense of achievement following the completion of the intervention.

Previous meta-analyses of randomized controlled trials have shown regular exercise, particularly PRT, or exercise plus nutrition approaches, are effective for improving muscle mass, strength, and performance in older adults with sarcopenia (13, 14). Our intervention was effective in improving muscle strength in both groups, but there were no significant changes in muscle mass. Participants reported completing most of their exercise sessions as we had the COVID-19 pandemic movement control order in Malaysia occur early in the intervention and had to revert to letting participants exercise at home. Participants mainly came to our campus to revise their exercise prescriptions based on their progress and to obtain WP supplementation thereafter. Since the number of enrolled and completed participants in this study was small (*n* = 16), the gains in muscle strength and changes in sarcopenia criterion may be considered case-study findings. Significant muscle strength changes were seen for handgrip values in both groups, and this may be attributed to the action of holding the resistance bands in a grip-like manner. Stair ascent time improved significantly by 0.8–1.0 s in both groups and 5-STS time by 1.3–2.0 s, which was significant for the PRT group. Thus, other than knowing which type of exercise works for sarcopenic older adults, participants' insights on how it may have worked for them are key to implementation.

Lack of knowledge on the health benefits of nutrition and exercise in older adults was a barrier to participation, as identified in a sarcopenia-related study (29). This was also evident in this study where participants identified the intervention as a preventive measure on top of managing sarcopenia. Participants wanted more guidelines and information on nutrition and recommended exercises to prevent or treat sarcopenia to be disseminated to them and their community. Information dissemination and awareness of sarcopenia are vital, and this includes having the medical fraternity be able to identify sarcopenia in their older patients (30). However, it remains to be explored how such information can be shared effectively and more widely. Future research could consider using digital tools and platforms and educating medical and health professionals about the importance of screening and diagnosing sarcopenia and how it can be treated. Another possible strategy is to apply implementation research or knowledge-translation study designs to assess the delivery of effective, scientific findings to the targeted community (31).

Reasons for non-participation in physical activity and exercise commonly include sociodemographic factors, including ethnicity, education level, and employment status (17). However, women in our study did not refer to their sociodemographic background as the main reason for not being physically active prior to participating in the research. Rather, it was the knowledge of their muscle condition or ability, i.e., sarcopenia, that largely determined their participation. Correspondingly, they focused on the perceived benefits that were achievable from participating in the intervention. Other concerns regarding participating in the exercise include lack of confidence and fear of falling or getting hurt, which are very real and are associated with less independent mobility and increased frailty in older adults (32, 33) and this was echoed by our participants. However, participants in this study were motivated to complete the intervention because they experienced positive change and felt better physically. Quantitatively, the change in stair ascent time was statistically significant and is not a criterion in sarcopenia but may be reflective of how these women feel after the intervention period as they could climb up the stairs at a faster pace. These self-reported improvements or patient-reported outcomes described the context of meaningful change that may be more relevant to people with sarcopenia (34). Overall reasons for participating and continuing with the intervention echoed the Health Belief Model along with other health behavior and social cognitive theories (35), where health motivation and perceived benefits (professional-guided PRT and suitable nutrition supplement) superseded their perceived barriers (fears and doubts) in managing sarcopenia. Through this study, we could see that we have unintentionally addressed components within behavioral change models to witness the change in participants' physical activity levels.

Both physical activity and nutrition are crucial in maintaining skeletal muscle mass and function in older adults (13, 14, 36). There is increasing research recognizing the critical role of increased dietary protein or protein supplements in combination with physical activity to prevent sarcopenia (37–40). Although milk consumption among older adults in Malaysia is considered uncommon (41), the high-protein whey-based supplement drink used in this study was well accepted due to its taste and limited side effects. Although there were no differences between PRT+WP and PRT groups, which is likely due to the small sample size, we also did not account for habitual dietary intake in this study and could not establish if either group had sufficient or inadequate nutrient intake. However, the MNA scores improved in the PRT+WP group, which also had more participants within the at-risk of malnutrition category at baseline. However, due to the small sample numbers, these findings are case-study-level evidence at best. The main messages from the personal perceptions of the participants were that: (1) the compliance with the nutritional supplement in this study was high (90%) and (2) the women enjoyed consuming the drink, which suggests that such an approach can be used to increase protein intake in older Malay women. Older adults can be encouraged to try whey-based products as an alternative to whole-milk products to minimize unpleasant experiences with normal milk intake and thus add good-quality proteins and nutrients to their diet.

Participants in this research who completed their exercise program at home indicated that they would prefer to attend a group-based, community program rather than exercising entirely by themselves at home, but they did not discuss any potential barriers or support needed to attend such programs. Barriers to attending a community-based program should be a focus area in future research as factors such as affordability and costs related to fees for exercise professionals to conduct programs were barriers to attending exercise sessions in several previous interventions (42, 43). However, research elucidating the types of exercise interventions for older adults remains inconclusive as the setting (clinical vs. community vs. home-based), qualified professional supervision, multimodal program components, or even group vs. individual-based program components influence long-term adherence, sustainability, and preferences across different populations (44). Researchers have highlighted the social aspect of a group program as a powerful driver for adherence and future continuation of being physically active (44), which is consistent with some of the findings from our study.

Other than health benefits, engagement in physical activity in older adults stemmed from feelings of being included; social engagement and being part of something that is accessible were major components as addressed by older adults in the literature (45). Our participants highlighted their preference to continue the PRT and WP programs in a community-based setting that evokes a similar sense of inclusiveness and social bonding through the activity. More specifically, participants were willing to allocate specific time to exercise and were motivated to continue with the exercises beyond the intervention. In this study, words such as “happy,” “blessed,” and “excited” were used to describe how they felt and were associated with how they perceived improvements to their physical health. Positive emotions can be enhanced and sustained by focusing on community-based physical activities as previous research has shown that community settings result in the enjoyment of physical exercises and positive emotions from the consequences of social networking (38). Participants' eagerness and motivation to engage were also evident in our study, which is crucial to ensure sustained engagement (46).

Sustained participation is related to having the opportunity to form social bonds and working closely with peers that will encourage and motivate one another (29), which was also mentioned by our participants. One study reported that some ethnic groups often focus on cultural sensitivity; for example, some Muslim women would not exercise in groups with men due to religious practices requiring gender segregation (42). However, this was not highlighted as an issue in our study, despite the fact that all were Muslim women. There is an interest in gender-specific differences in the development of sarcopenia (29), and although participants were very keen to change their behavior to be healthier with the strong advocation for structured exercise sessions, our study is limited to views from women only as no men participated in this study.

Encouraged by the findings from this pilot trial of the PRT and WP interventions that were both quantitatively and qualitatively analyzed, a more comprehensive implementation type of research is required. Note that our study results were limited to views and physiological responses from a small all-women cohort, and the results should be interpreted with caution. Furthermore, during the study, we had challenges with access and logistics due to the COVID-19 movement control orders in Malaysia which may have reduced our efficiency to effectively monitor and determine compliance with all the PRT sessions and supplementation intake. However, physical changes were evident, and participants would meet to assess progression, change exercise bands, and receive the next dose of the whey protein supplementation. Although we monitored participants via weekly phone calls, there may be self-report biases as one individual deteriorated to severe sarcopenia despite reporting completing the prescribed exercises and supplementation. Furthermore, our study only included one ethnicity (Malays) in Malaysia, and thus, future research should include multiple populations and settings to capture a more inclusive perception. Finally, the study design was geographically restricted to Malaysia's East Coast, but the methods can be replicated elsewhere to include wider sociodemographic factors and ultimately capture the views of the culturally diverse population in Malaysia.

## 5. Conclusion

In this study of older Malay women with sarcopenia, the overall physical outcomes were encouraging, and the experiences reflected upon by the women completing the 12 weeks of exercise and nutrition intervention were positive, with valuable recommendations to inform future interventions and in designing prevention activities within a community setting, especially in Malaysia. While the women were initially skeptical and fearful of participating in a multi-faceted exercise and nutrition program, they reported a positive experience following the intervention due to the perceived physical and mental health benefits. The changes they made to their lifestyle for 12 weeks as part of the intervention were something that they were willing to sustain in their daily lives with adequate support and guidance. However, to facilitate future implementation and participation, women indicated they want more multi-model exercises, nutrition guidance, and programs that are incorporated into community-based settings. Collectively, the findings from this study indicated that engagement in physical activities and subsequently preventing sarcopenia among older adults can be successful with the right support, facilities, and guidance that can be investigated within an implementation research design.

## Data availability statement

The raw data supporting the conclusions of this article will be made available by the authors, without undue reservation.

## Ethics statement

The studies involving human participants were reviewed and approved by Human Research Ethics of Universiti Sains Malaysia. The patients/participants provided their written informed consent to participate in this study.

## Author contributions

VT conceptualized, conducted formal analysis and investigation of the study, drafted, and reviewed the manuscript. RV performed investigation and data analyses, prepared original draft of the manuscript, reviewed, and edited the manuscript. RD provided research resources, reviewed, and edited the manuscript. All authors read and approved the final manuscript.
