# Supplementary material for: “We want more”: perspectives of sarcopenic older women on the feasibility of high-intensity progressive resistance exercises and a whey-protein nutrition intervention
Source: Front Nutr. 2023 Sep 7;10:1176523. doi: 10.3389/fnut.2023.1176523 (PMC10513027; doi:10.3389/fnut.2023.1176523)
Supplement: Supplementary file 1 [file Data_Sheet_1.PDF]

Additional File 1: Interview guide for progressive high-intensity resistance training (PRT) and whey-protein complete nutrition (CN) supplementation intervention study for older people with sarcopenia.

Interview scope and guide questions.

1. What are the factors considered by older Malaysian adults with sarcopenia when taking part in PRT?

2. What are the factors considered by older Malaysian adults with sarcopenia in consuming a CN supplement?

3. What are the motivation factors or barriers to participate in PRT in older Malaysian adults with sarcopenia?

4. What are the motivation factors or barriers to consume a CN supplement in older Malaysian adults with sarcopenia?

5. What are the perceptions of older Malaysian adults with sarcopenia about the PRT?

6. What are the perceptions of older Malaysian adults about the CN supplement consumed?
