# Supplementary material for: “We want more”: perspectives of sarcopenic older women on the feasibility of high-intensity progressive resistance exercises and a whey-protein nutrition intervention
Source: Front Nutr. 2023 Sep 7;10:1176523. doi: 10.3389/fnut.2023.1176523 (PMC10513027; doi:10.3389/fnut.2023.1176523)
Supplement: Supplementary file 2 [file Table_1.docx]

**Macro and micro-nutrient list of the whey-supplementation**

| **Nutrients** | **Per serving (57g)** | **Per 100g** |
| --- | --- | --- |
| **Energy (kcal)** | 237 | 416 |
| **Protein (g)** | 15.1 | 26.5 |
| **Fat** | |  |
| Total fat (g) | 7.0 | 12.2 |
| Monosaturated fat (g) | 4.7 | 8.3 |
| Polyunsaturated fat (g) | 0.7 | 1.2 |
| Saturated fat (g) | 1.4 | 2.5 |
| Trans-fat (g) | 0.1 | 0.2 |
| **Carbohydrate** | |  |
| Total carbohydrate (g) | 26.7 | 46.8 |
| Sugars (g) | 3.1 | 5.4 |
| Dietary fibre (g) | 3.1 | 5.4 |
| **Amino Acid** |  |  |
| Aspartic acid (g) | 1.4 | 2.40 |
| Serine (g) | 0.9 | 1.60 |
| Glutamic acid (g) | 2.2 | 3.92 |
| Glycine (g) | 0.3 | 0.46 |
| Histidine (g) | 0.3 | 0.49 |
| Arginine (g) | 0.4 | 0.72 |
| Threonine (g) | 1.1 | 1.96 |
| Alanine (g) | 0.7 | 1.19 |
| Proline (g) | 0.9 | 1.62 |
| Tyrosine (g) | 0.4 | 0.65 |
| Valine (g) | 0.9 | 1.50 |
| Methionine (g) | 0.3 | 0.51 |
| Lysine (g) | 1.2 | 2.09 |
| Isoleucine (g) | 0.9 | 1.55 |
| Leucine (g) | 1.6 | 2.87 |
| Phenylalanine (g) | 0.5 | 0.90 |
| Tryptophan (g) | 0.2 | 0.42 |
| Cysteine (g) | 0.2 | 0.43 |
| **Vitamins** | |  |
| A (mcg) | 188 | 330 |
| B1, Thiamin (mg) | 0.4 | 0.6 |
| B2, Riboflavin (mg) | 0.5 | 0.9 |
| B3, Niacin (mg) | 3.6 | 6.3 |
| B5, Panthothenic acid (mg) | 0.8 | 1.4 |
| B6, Pyridoxine (mg) | 0.7 | 1.2 |
| B7, Biotin (mcg) | 5.0 | 8.8 |
| B9, Folate (mcg) | 70 | 123 |
| B12, Cobalamin (mcg) | 0.7 | 1.2 |
| C (mg) | 20 | 35 |
| D3 (mcg) | 188 | 7.0 |
| E (mg) | 4.0 | 8.8 |
| K1 (mcg) | 20 | 35 |

| **Nutrients** | **Per serving (57g)** | **Per 100g** |
| --- | --- | --- |
| **Minerals** | |  |
| Calcium (mg) | 250 | 439 |
| Iodine (mcg) | 38 | 67 |
| Selenium (mcg) | 16 | 28 |
| Zinc (mg) | 4.0 | 7.0 |
| Iron (mg) | 3.3 | 5.8 |
| Phosphorus (mg) | 126 | 221 |
| Sodium (mg) | 216 | 379 |
| Potassium (mg) | 386 | 677 |
| Magnesium (mg) | 58 | 102 |
| Chromium (mcg) | 30 | 53 |
| Copper (mcg) | 300 | 527 |
| Manganese (mg) | 0.7 | 1.2 |
| Molybdenum (mcg) | 26 | 46 |
| Chloride (mg) | 240 | 422 |
